# Research note changes in the number of outpatient visits in Japan during the COVID-19 pandemic

**DOI:** 10.1186/s13104-023-06336-9

**Published:** 2023-05-04

**Authors:** Munehito Machida, Yuichi Nishioka, Tatsuya Noda, Tomoaki Imamura

**Affiliations:** 1 Department of Public Health, Health Management and Policy, Nara Medical University, 840 Shijo-Cho, Kashihara city, 634-8521 Nara Japan; 2 Department of Public Health Policy, National Institute of Public Health, Wako city, Saitama Japan

**Keywords:** Outpatient visits, COVID-19, Infectious diseases, Chronic diseases

## Abstract

**Objective:**

This study aimed to determine the change in the number of outpatient visits in Japan since the beginning of the COVID-19 pandemic, using data on the outpatient claims submitted by medical institutions to insurers in 2019 and 2020, from the National Database of Health Insurance Claims and Specific Health Check-ups. We calculated the total number of outpatient visits, and number of visits for individual diseases according to the International Classification of Diseases-10 codes on the claim form.

**Results:**

The number of outpatient visits per million people decreased by 9.98% in 2020 compared to 2019. Of the diseases included in the analysis, 71 showed a decrease in the number of visits by ≥ 1%. There were significant decreases in the number of visits related to infectious diseases (influenza, acute bronchitis, and acute laryngitis, etc.), and chronic diseases (hemorrhoids, cystic kidney disease, dyspepsia, and chronic sinusitis, etc.). The observed decreased rate of outpatient visit might have been due to, a decrease in the incidence of disease, a decreased frequency of visit by patients with the disease, or both. Our analysis method using actual health insurance claim data can be applied worldwide, where researchers have access to national information on health insurance claims.

## Introduction

The annual number of outpatient visits in Japan is 12.4 per capita, which is relatively high compared to other Organization for Economic Co-operation and Development (OECD) countries [1]. Japan has a universal health insurance system for its population and within the framework of this system, the country has meticulously established a reimbursement system for high-cost medical care, and exemptions for individuals with limited income, which allows the public free access to medical care, both as policy and in practice. However, the frequency of outpatient visits reportedly decreased during the COVID-19 pandemic due to fear of getting infected [2]. Therefore, this study aimed to determine the change in the number of outpatient visits in Japan since the beginning of the COVID-19 pandemic using a comprehensive administrative database containing data of almost 100 million Japanese residents.

## Methods

The data were obtained from the National Database (NDB) of Health Insurance Claims and Specific Health Check-ups [3]. This database contains data collected using standardized health insurance claim forms used by medical facilities for claiming reimbursement from insurers. The insurance claims of almost all citizens are digitized in the same format, regardless of the insurer type [1]. The Ministry of Health, Welfare and Labor of Japan developed the NDB for planning, evaluating, and optimizing medical costs. The NDB data are available for analysis to a limited number of eligible applicants, including national administrative agencies, prefectures, universities, and national level medical insurers [4]. As of March 2019, 99.2% of hospitals, and 94.8% of clinics in Japan had digitized all health insurance claims, making research using data from the NDB more accessible across the nation [5].

Based on the outpatient claims submitted monthly by medical institutions to insurers in 2019 and 2020, we calculated the total number of outpatient visits, and number of visits for each disease according to the International Classification of Diseases-10 (ICD-10) codes on the claim form. The number of visits was converted to units per million population using the following formulae:

$$\begin{array}{l}{\rm{Visit\ rate\ per\ million\ population\ per\ year}} = \\{\rm{(total\ number\ of\ outpatient\ visits)}}\\\div {\rm{(total\ population\ based }}\\{\rm{on\ the\ Basic\ Resident\ Ledger\ in\ 2020)}} \times 365 \times {10^6}\end{array}$$

$$\begin{array}{l}{\rm{Visit\ rate\ per\ million\ population\ per\ year\ by\ disease }} = \\{\rm{(total\ number\ of\ outpatient\ visits\ for\ the\ disease)}}\\\div {\rm{(total\ population\ based }}\\{\rm{on\ the\ Basic\ Resident\ Ledger\ in\ 2020)}} \times 365 \times {10^6}\end{array}$$

If a patient received treatment for multiple diseases during a single visit, each treatment was counted as a visit for that particular disease. Diseases for which the number of outpatient visits increased from 2019 to 2020 received a negative score.

The difference in the number of visits in 2019 and 2020 was set as the denominator, and the difference in the number of visits for each disease in 2019 and 2020 was set as the numerator. From this, we calculated the percentage decrease in the visit rate per disease using the formula:

$$\begin{array}{l}{\rm{Percentage\ decrease\ in\ the }}\\{\rm{visit\ rate\ for\ the\ disease}}\\{\rm{ = 1 - (visit\ rate\ for\ the\ disease\ in\ 2020)}}\\\div {\rm{(visit\ rate\ for\ the\ disease\ in\ 2019)}} \times {\rm{100 }}\end{array}$$

This study complied with the provisions of the World Medical Association Declaration of Helsinki (as amended by the 59th General Assembly, Seoul, the Republic of Korea, October 2008), and was approved by the Ethical Review Committee of Nara Medical University (No.: 1123). The requirement for informed consent was waived as the data were anonymized.

## Results

The number of outpatient visits per million people decreased by 9.98%, from 33,466 to 2019 to 30,127 in 2020. Of all the diseases included in the analysis, 71 showed a decrease in the number of visits by ≥ 1% (Table 1). There were significant decreases in the number of outpatient visits related to infectious diseases (such as influenza, viral infections of the skin, and mucous membranes, acute bronchitis, acute laryngitis, and acute upper respiratory tract infection), and chronic diseases (such as hemorrhoids, cystic kidney disease, dermatitis due to substances taken internally [ICD-10 code L27], dyspepsia, chronic sinusitis, and asthma).

**Table 1:** Diseases with significant decreases in outpatient visits

ICD10	Name of Disease	Average number of outpatient visits in one day (per million)		Proportion of decrease from 2019 to 2020 (%)
		2019	2020	
K64	Hemorrhoids and perianal venous thrombosis	610	145	76.17
J10	Influenza due to other identified influenza virus	313	91	71.08
J11	Influenza, virus not identified	156	49	68.85
Q61	Cystic kidney disease	305	100	67.38
L27	Dermatitis due to substances taken internally	277	102	63.23
B08	Other viral infections characterized by skin and mucous membrane lesions, not elsewhere classified	111	53	52.06
K30	Functional dyspepsia	200	107	46.70
J20	Acute bronchitis	1,960	1,063	45.78
J04	Acute laryngitis	140	81	41.80
J06	Acute upper respiratory infections of multiple and unspecified sites	2,300	1,379	40.03
J40	Bronchitis, not specified as acute or chronic	494	302	38.91
J01	Acute sinusitis	595	371	37.74
J03	Acute tonsillitis	332	210	36.96
J02	Acute pharyngitis	1,200	757	36.91
H66	Suppurative and unspecified otitis media	290	185	36.35
I67	Other cerebrovascular diseases (dissection of cerebral arteries [non-ruptured], cerebral aneurysm [non-ruptured], cerebral atherosclerosis, etc.)	478	306	36.00
J00	Acute nasopharyngitis (common cold)	647	423	34.71
H65	Nonsuppurative otitis media	244	171	29.97
J18	Bronchopneumonia, unspecified	238	168	29.58
J32	Chronic sinusitis	881	677	23.17
R50	Fever of other and unknown origin	225	173	23.06
A09	Other gastroenteritis and colitis of infectious and unspecified origin	1,368	1,061	22.46
E86	Volume depletion	495	386	21.96
A49	Bacterial infection of unspecified site	367	292	20.24
J45	Asthma	2,617	2,097	19.87
J31	Chronic rhinitis, nasopharyngitis and pharyngitis	231	191	17.38
J30	Vasomotor and allergic rhinitis	4,952	4,100	17.22
H61	Other disorders of external ear (Perichondritis of external ear, non-infective disorders of pinna, impacted cerumen, etc.)	268	225	16.10
R11	Nausea and vomiting	622	544	12.49
H60	Otitis externa	392	344	12.33
H10	Conjunctivitis	2,504	2,210	11.71
K12	Stomatitis and related lesions	418	379	9.53
K25	Gastric ulcer	1,873	1,705	8.95
M47	Spondylosis	3,051	2,792	8.50
H26	Other cataract (infantile, juvenile, and presenile cataract; traumatic cataract; drug-induced cataract; etc.)	961	880	8.46
T14	Injury of unspecified body region	1,005	922	8.28
M17	Gonarthrosis (arthrosis of knee)	3,173	2,911	8.24
H53	Visual disturbances	416	382	8.18
K29	Gastritis and duodenitis	5,760	5,290	8.15
G62	Other polyneuropathies (drug-induced polyneuropathy, alcoholic polyneuropathy, etc.)	1,626	1,498	7.91
M48	Other spondylopathies (spinal stenosis, ankylosing hyperostosis, kissing spine, etc.)	1,771	1,636	7.59
H52	Disorders of refraction and accommodation	2,688	2,491	7.34
I63	Cerebral infarction	960	890	7.32
R42	Dizziness and giddiness	913	848	7.08
M81	Osteoporosis without pathological fracture	3,554	3,307	6.96
M53	Other dorsopathies, not elsewhere classified (cervicocranial syndrome, cervicobrachial syndrome, spinal instabilities, etc.)	1,009	940	6.89
I69	Sequelae of cerebrovascular disease	716	669	6.56
M75	Shoulder lesions	1,950	1,822	6.55
H16	Keratitis	745	697	6.40
M51	Other intervertebral disc disorders (lumbar and other intervertebral disc disorders with myelopathy or radiculopathy, other specified intervertebral disc displacement, etc.)	867	812	6.35
I20	Angina pectoris	1,972	1,848	6.27
R51	Headache	855	802	6.22
G98	Other disorders of nervous system, not elsewhere classified	766	718	6.19
L50	Urticaria	578	543	6.00
M54	Dorsalgia	4,344	4,084	5.98
I49	Other cardiac arrhythmias (ventricular fibrillation and flutter, atrial premature depolarization, sick sinus syndrome, etc.)	930	875	5.95
M13	Other arthritis (polyarthritis [unspecified], monoarthritis [not elsewhere classified], etc.)	643	606	5.86
E14	Unspecified diabetes mellitus	3,170	2,984	5.86
I70	Atherosclerosis	1,176	1,107	5.81
J42	Unspecified chronic bronchitis	823	782	4.99
G47	Sleep disorders	4,505	4,309	4.35
M79	Other soft tissue disorders, not elsewhere classified (rheumatism [unspecified], myalgia, fibromyalgia, etc.)	1,372	1,314	4.24
I10	Essential (primary) hypertension	8,747	8,400	3.97
L30	Other dermatitis (nummular dermatitis, dyshidrosis [pompholyx], cutaneous autosensitization, etc.)	3,171	3,046	3.92
K76	Other diseases of liver (fatty [change of] liver [not elsewhere classified], chronic passive congestion of liver, central hemorrhagic necrosis of liver, infarction of liver, etc.)	1,693	1,628	3.85
F41	Other anxiety disorders (panic disorder [episodic paroxysmal anxiety], generalized anxiety disorder, mixed anxiety and depressive disorder, etc.)	1,050	1,012	3.62
B35	Dermatophytosis	1,213	1,170	3.54
N40	Hyperplasia of prostate	1,040	1,005	3.34
E78	Disorders of lipoprotein metabolism and other lipidaemias	6,633	6,413	3.33
K59	Other functional intestinal disorders (constipation, functional diarrhea, neurogenic bowel [not elsewhere classified], etc.)	4,985	4,836	2.99
K21	Gastro-esophageal reflux disease	4,639	4,533	2.29

## Discussion

This study focused on the decline in outpatient visits during the COVID-19 epidemic. The observed decrease in the outpatient visit rate might have been due to a decrease in the incidence of disease, or a decreased frequency of visit by patients with the disease, or both. For instance, as genetic factors play a role in the development of some cancers, and cancers may develop over a period of years or decades, a certain percentage of the population will always be affected, regardless of the pandemic. The decrease in the visit rate for such diseases was probably due to an avoidance of medical consultations rather than a decrease in the disease incidence. On the other hand, for diseases that are strongly influenced by short-term changes in behavior, the decrease in the visit rate may have been either due to a decreased incidence of the disease, or an avoidance of medical consultations, or both. Previous studies have reported a decrease in the incidence of pediatric viral respiratory tract infections due to social distancing [4], and an absent seasonal influenza epidemic during the 2020–2021 influenza season across all age groups [5], demonstrating evidence of a decrease in the incidence of infectious diseases due to the pandemic. Access to medical care is likely to have become limited as a result of a combination of prioritization of care for COVID-19 patients, together with avoidance of consultation by patients for fear of contracting COVID-19. This could explain the reduction in the number of visits for chronic conditions. As this study analyzed data retrospectively, prospective studies can be designed to further validate our results.

As our dataset included more than 100 million people, and Japan has universal health insurance coverage, we were able to identify large-scale societal trends in medical consultations associated with the COVID-19 pandemic, such as a decrease in the incidence of infectious diseases other than COVID-19, a temporary limited access to medical facilities, and the avoidance of medical consultations.

Our analysis method using actual health insurance claim data can be applied not only in Japan but also in other countries where researchers have access to national information on health insurance claims [6–8]. As universal health coverage continues to be implemented in low- and middle-income countries, in keeping with World Health Organization recommendations, consideration should be given to developing health insurance databases also, which can be used for research purposes to draw-up health care policies not only for management of health crises such as the COVID-19 pandemic, but also for strengthening health care systems in general [9, 10].

Japan needs to consider ensuring that all patients have the right to receive necessary outpatient care during future pandemics and otherwise. In Japan, prior to the COVID-19 pandemic, telemedicine was covered by medical insurance only for very limited applications compared to other countries. For example, telemedicine was only allowed for re-consultation in underpopulated areas. During the COVID-19 epidemic in Japan, expansion of the use of telemedicine was actively discussed, and eventually initial telemedicine consultations for patients suspected of having COVID-19 in urban areas were included in the coverage by medical insurance on a limited basis [11]. We hope that Japan will review its policies with reference to reports regarding inclusion of medical insurance coverage of telemedicine consultations in other countries [12–14].

### Limitations

This study was unable to determine whether decreased incidence of disease or decreased access to care predominated in causing the decrease in the number of outpatient visits during COVID-19 pandemic. The underlying cause is likely to have varied from disease to disease.

## Data Availability

The datasets analyzed during the current study are available from the corresponding author on reasonable request.

## Abbreviations

OECD: Organization for Economic Co-operation and Development
NDB: National Database
ICD-10: International Classification of Diseases-10
